# Triple tendon transfer for an irreparable 10-cm radial nerve defect: a case report of functional recovery and a successful return to work as a waiter

**DOI:** 10.1097/RC9.0000000000000764

**Published:** 2026-08-07

**Authors:** Ilhame Khmilech, Salissou N.H. Saidou, Walid Bouziane, Mohammed Sadougui, Abdelkrim Daoudi

**Affiliations:** a Orthopedics and Trauma Department A, Mohammed VI University Hospital, Oujda, Morocco; b Faculty of Medicine and Pharmacy Oujda, Mohammed First University, Oujda, Morocco

**Keywords:** case report, functional recovery, nerve gap, radial nerve palsy, tendon transfer

## Abstract

**Introduction:**

Radial nerve palsy following humeral shaft fractures is common, but management of massive nerve gaps remains a challenge. While nerve grafting is an option, defects exceeding 10 cm often yield poor results.

**Case presentation:**

We report the case of an 18-year-old male, a chronic smoker, who sustained a complete 10-cm radial nerve loss; management consisted of a triple tendon transfer.

**Results:**

At 14 months, he achieved complete extension and successfully returned to his demanding professional activity.

**Discussion:**

The use of the flexor carpi ulnaris for digital and thumb extension, combined with transfer of the pronator teres to the extensor carpi radialis brevis for wrist balance, allowed restoration of both power and stability. These results support tendon transfer as a preferred option for large nerve defects when early functional recovery is the priority.

**Conclusion:**

The patient’s smoking status, combined with a 10-cm nerve gap, favored tendon transfer as a reliable option for immediate and predictable functional restoration in massive radial nerve defects.

## Introduction

The radial nerve is the most frequently injured peripheral nerve in long bone fractures, with an incidence ranging from 2% to 17% in humeral shaft injuries^[^1–3^]^. While approximately 70% to 90% of cases involving closed fractures achieve spontaneous recovery through expectant management, high-energy trauma often results in neurotmesis (Sunderland Grade V)^[^1,2^]^.

When surgical exploration reveals a massive nerve defect, specifically exceeding the 8-cm threshold, nerve grafting often yields disappointing results for the recovery of distal extension of the fingers and thumb^[^1,4^]^. In such cases, or when the “biological window” for reinnervation (typically 12–18 months) has closed, palliative tendon transfer (TT) becomes the primary indication for functional restoration^[^2,4,5^]^. Unlike nerve-based procedures, TTs provide an immediate mechanical solution that is reproducible and independent of the biological constraints of axonal regeneration^[^4,5^]^.

HIGHLIGHTS

- A 10-cm radial nerve gap was successfully managed with a definitive triple tendon transfer via a four-incision approach.
- The choice of the flexor carpi ulnaris as a donor provides superior biomechanical power, essential for high-demand manual professions.
- Preservation of wrist balance was achieved by transferring the pronator teres solely to the extensor carpi radialis brevis, effectively mitigating the risk of radial deviation.
- The patient achieved complete functional restoration at 14 months, enabling a successful return to professional activity as a waiter.

This case has been reported in accordance with the updated SCARE 2025 criteria^[^6^]^.

## Case presentation

### Initial presentation and management

An 18-year-old right-handed male, a chronic smoker, presented following a high-energy motorcycle accident. Clinical examination revealed a classic drop-wrist deformity of the dominant hand, with a total loss of wrist, finger, and thumb extension. Radiographs confirmed a displaced right humeral shaft fracture. Initial management consisted of open reduction and internal fixation with a dynamic compression plate. During this initial procedure, the radial nerve was found to be completely sectioned at the level of the fracture, with a 10-cm gap. Given the length of the defect, the patient’s smoking status (which potentially slows nerve regeneration) and his requirement for a rapid return to manual labor, a definitive triple TT was chosen over nerve grafting^[^4,7^]^.

### Surgical technique

**Figure 1.**
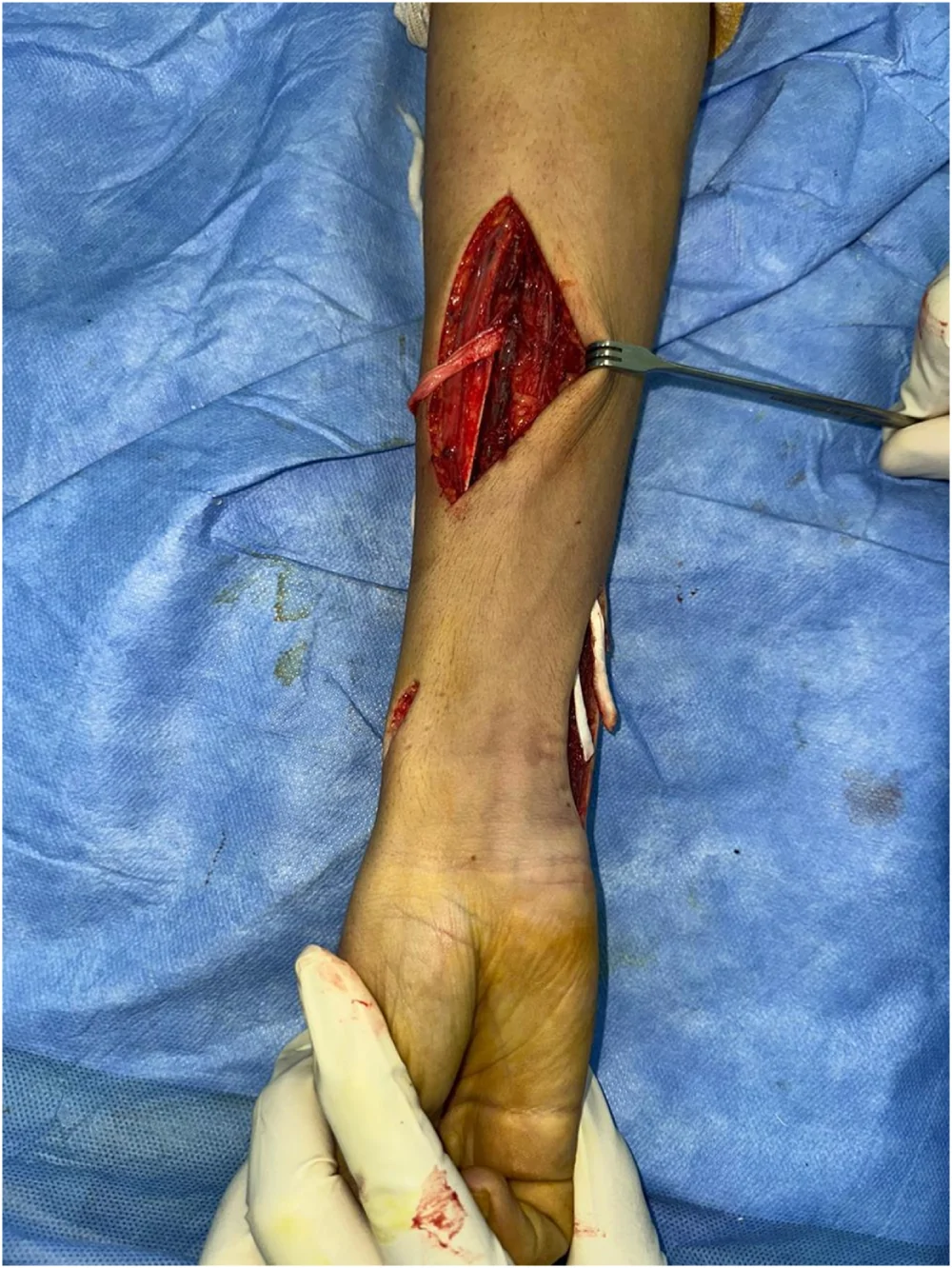
A multi-incision approach was used to harvest the donor tendons: the pronator teres, palmaris longus, and flexor carpi ulnaris.

**Figure 2.**
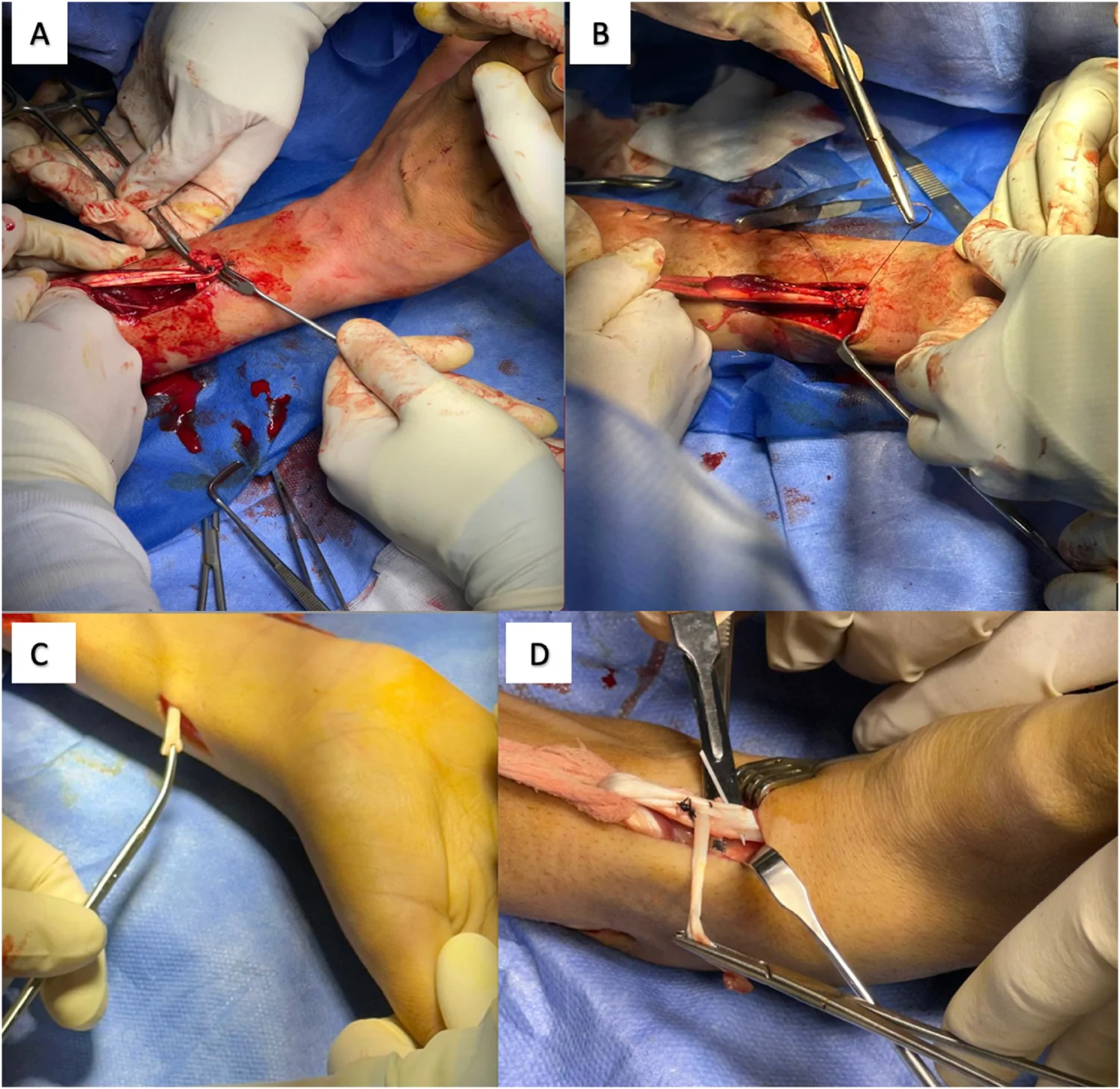
The flexor carpi ulnaris was sutured to the extensor digitorum communis and extensor pollicis longus (A) using a pulvertaft weave technique (B); then the palmaris longus was retrieved (C) and sutured (D) to the abductor pollicis longus to restore the opening of the first web space.

Under general anesthesia, a modified Merle d’Aubigné and Jones triple transfer was performed using a four-incision approach:

1. Wrist extension: Through a proximal volar forearm incision, the Pronator Teres (PT) was harvested from the radius with a 3-cm periosteal strip to ensure adequate length to reach (Fig. 1). It was transferred to the extensor carpi radialis brevis (ECRB).
2. Finger and thumb extension: A distal ulnar incision allowed the harvest of the flexor carpi ulnaris (FCU) and the palmaris longus (PL) (Fig. 1).
3. A dorsal wrist incision allowed tendon passage: the FCU was transferred to the extensor digitorum communis and the extensor pollicis longus (Fig. 2).
4. Thumb abduction: A separate incision at the base of the thumb (first extensor compartment) was used to isolate the abductor pollicis longus, to which the PL was transferred (Fig. 2).

Coaptations were performed proximal to the extensor retinaculum using the Pulvertaft weave technique. Tensioning was carefully adjusted to respect the physiological descending finger cascade, while the wrist was maintained at 40° of extension (Fig. 2).

**Figure 3.**
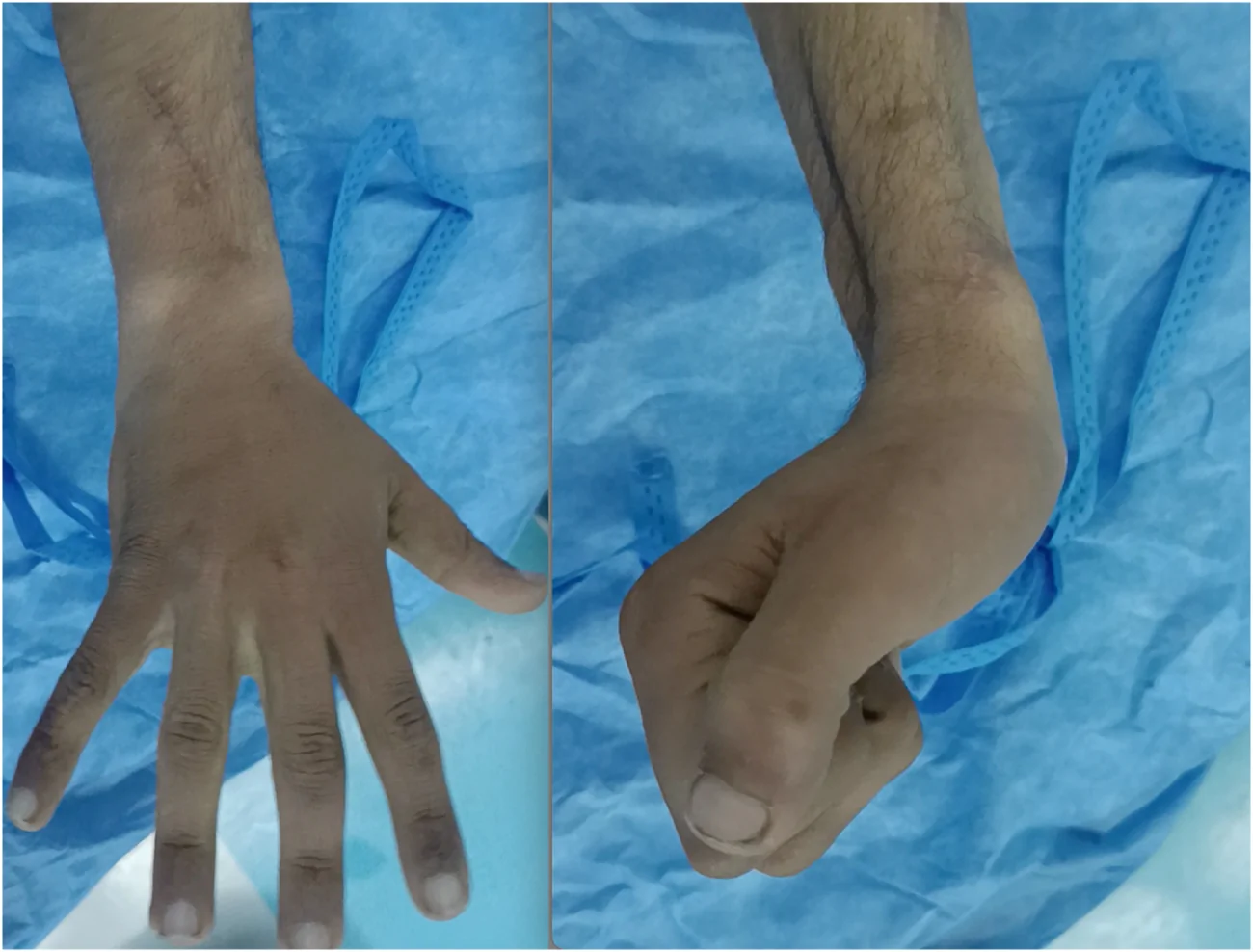
Clinical follow-up at 14 months, demonstrating complete active extension of the wrist and all digits.

Postoperative evolution: Following a rigorous rehabilitation program focused on muscle retraining, as shown in Table 1, the patient showed progressive improvement. At 14-month follow-up, he demonstrated complete active extension of the wrist and all digits (Fig. 3), with full consolidation of the humeral fracture confirmed; he also reported a successful return to his professional activities as a waiter in a café, a role requiring significant grip strength and manual stability for carrying heavy trays.

**Table 1:** Graduated postoperative rehabilitation protocol following triple tendon transfer.

Phase	Timeline	Treatment goals and interventions
Protective	0–4 weeks	Immobilization: Brachial-palmar splint (wrist 40–50° ext, MCPJ 0°, thumb full abd). Edema control and wound healing.
Initial active	4–6 weeks	Protected motion: Change to short-arm splint. Gentle active flexion and passive extension. Start active-assisted ROM.
Muscle retraining	6–8 weeks	Re-education: Synergistic exercises (e.g., wrist extension during forearm pronation). Wean from daytime splinting.
Strengthening	8–12 weeks	Loading: Progressive resistance exercises. Hand function training (grasp/release). Night splinting continued.
Return to work	>12 weeks	Full activity: Heavy load training (tray carrying simulation). Resumption of professional duties.

## Discussion

The management of irreparable radial nerve palsy remains a subject of debate, particularly regarding the choice between nerve reconstruction and TTs^[^1^]^. Our case highlights several key factors that justify the use of TTs as a primary reconstructive strategy for massive nerve gaps.

1. The 10-cm Gap: The Limit of Nerve Grafting

The most critical factor in our clinical decision was the 10-cm nerve defect. While nerve grafts can sometimes succeed, the literature consistently shows that results for distal motor recovery decline sharply for gaps exceeding 8 cm. A massive pooled analysis of 754 patients by Jain et al. (2024) demonstrated that TTs achieved “good” outcomes in 82% of cases, significantly outperforming nerve grafts (32%) and nerve transfers (59%)^[^5^]^. TTs bypass the 12–18 month “biological deadline” for motor endplate reinnervation, offering guaranteed mechanical stability^[^2,5^]^.

2. Donor Selection: FCU for High-Demand Manual Labor

A key technical choice was to use the FCU for finger and thumb extension, a hallmark of the Merle d’Aubigné technique^[^4,8,9^]^.

- Biomechanical Power: The FCU is a powerful motor with a strength of approximately 2 kg, compared with only 1.1 kg for the FCR^[^4,10^]^.
- Functional Requirement: For a patient working as a waiter, grip strength is paramount. Studies indicate that FCU-based transfers can restore grip strength to approximately 65% of the healthy side^[^9^]^.
- Mitigating Radial Deviation: While some authors warn that harvesting the FCU may lead to radial deviation^[^10,11^]^, we mitigated this risk by transferring the PT solely to the ECRB^[^4,10,11^]^. The central insertion of the ECRB on the third metacarpal provides a balanced extension that counteracts frontal plane imbalance^[^4,10^]^.

3. Technical Nuances: The Periosteal Strip and Pulvertaft Weave

The success of the PT-to-ECRB transfer is highly dependent on tension and length^[^4,9^]^. Harvesting the PT with a periosteal strip, as performed in our patient, is a vital maneuver to ensure a robust weave^[^11,12^]^. Furthermore, our use of the Pulvertaft weave proximal to the extensor retinaculum prevented tendon bowstringing and preserved maximal excursion^[^13,14,15^]^. Although PT transfer can lead to a 24%–44% loss of pronation strength^[^16^]^, our patient, like many in recent series, reported no functional deficit in professional pronation^[^4^]^.

4. Prognostic Factors: Age and Professional Reintegration

The patient’s young age (18 years) was a significant positive predictor, facilitating rapid cortical adaptation and “muscle retraining”^[^2,4,10^]^. At 14 months, the patient’s return to full-time work as a waiter confirms the functional success of the procedure. According to current outcome scales, the achievement of complete active extension and a high level of patient satisfaction justifies this approach as a primary choice for massive irreparable radial nerve palsy^[^1,9,10^]^.

5. Comparison with Established Series

Our results align with Ropars *et al*^[^10^]^ and Bertelli^[^17^]^, who reported that tendon-transfer patients often recover a functional arc of motion (approximately 34–38° extension and 35–41° flexion). Unlike cases described by Richford *et al*^[^13^]^, where some patients defaulted on therapy and experienced stiffness, our patient’s young age (18 years) and high motivation for professional reintegration facilitated rapid cortical adaptation and a good functional outcome.

## Conclusion

For irreparable radial nerve injuries with a massive 10-cm gap, triple TT remains the most reliable and reproducible technique^[^1^]^. It provides immediate mechanical stability and superior strength compared to nerve grafting, allowing for a full return to professional life even in high-demand manual occupations^[^1,4^]^. Surgeons should prioritize the ECRB for wrist balance and consider the FCU a viable and powerful donor when high grip strength is required postoperatively^[^1,9,10^]^.

## Data Availability

The data supporting this study are available from the corresponding author upon reasonable request.

## References

[1] Korompilias AV Lykissas MG Kostas-agnantis IP. Approach to radial nerve palsy caused by humerus shaft fracture: is primary exploration necessary? Injury 2013;44:323–326. 10.1016/j.injury.2013.01.004 23352153

[2] Daly M Langhammer C. Radial nerve injury in humeral shaft fracture USA. Ortho Clin North America 2022;53:145–154. 10.1016/j.ocl.2022.01.001 35365259

[3] Bumbasirevic M Palibrk T Lesic A. Radial nerve palsy. EFORT Open Rev 2016;1:286–294. 10.1302/2058-5241.1.000028 PMC5367587 28461960

[4] Tordjman D D’utruy A Bauer B. Tendon transfer surgery for radial nerve palsy. Hand Surg Rehab 2022;41:S90–S97. 10.1016/j.hansur.2018.09.009 34343724

[5] Jain NS Barr ML Kim D. Tendon transfers, nerve grafts, and nerve transfers for isolated radial nerve palsy: a systematic review and analysis. Hand (New York, N,Y) 2024;19:343–351. 10.1177/15589447221150516 PMC11067830 36692098

[6] Agha RA Mathew G Rashid R. Revised preferred reporting of case series in surgery (PROCESS) guideline: an update for the age of artificial intelligence. Prem J Sci 2025. doi:10.70389/PJS.100079

[7] Wang S Zhou W Zhou Q. Comparison of nerve versus tendon transfer for radial nerve palsy. Clin Neurol Neurosurg 2024;236:108077. 10.1016/j.clineuro.2023.108077 38091705

[8] Nachef N Bariatinsky V Sulimovic S. Predictors of radial nerve palsy recovery in humeral shaft fractures: a retrospective review of 17 patients. Ortho Traumatol 2017;103:177–182. 10.1016/j.otsr.2016.10.023 28065869

[9] Mai TT Nguyen VQ Nguyễn PD. Treatment of irrecoverable radial nerve palsy using the modified merle d’aubigné tendon transfer method. Orthop Rev (Pavia) 2024;16. doi:10.52965/001c.94033 PMC10891145 38404927

[10] Ropars M Dréano T Siret P. Long-term results of tendon transfers in radial and posterior interosseous nerve paralysis. J Hand Surg 2006;31:502–506. 10.1016/j.jhsb.2006.05.020 16928411

[11] Besnard M Marteau E Laulan J. Tendon transfers for radial nerve palsy with extensor carpi ulnaris revival: technique and results. Ortho Traumatol 2020;106:307–310. 10.1016/j.otsr.2019.11.026 32061574

[12] Tsuge K. Tendon transfers for radial nerve palsy. Australian New Zealand J Surg 1980;50:267–272. 10.1111/j.1445-2197.1980.tb04115.x 6931587

[13] Department of Orthopaedics and Traumatology, Universiti Kebangsaan Malaysia, Cheras, Malaysia. Outcome of tendon transfers for radial nerve palsy in a malaysian tertiary centre. MOJ 2018;12:1–6. 10.5704/MOJ.1803.001 PMC5920251 29725505

[14] Cheah AE-J Etcheson J Yao J. Radial nerve tendon transfers. Hand Clin 2016;32:323–338. 10.1016/j.hcl.2016.03.003 27387076

[15] Jones NF Machado GR. Tendon transfers for radial, median, and ulnar nerve injuries: current surgical techniques. Clin Plast Surg 2011;38:621–642. 10.1016/j.cps.2011.07.002 22032590

[16] Skie MC Parent TE Mudge KM. Functional deficit after transfer of the pronator teres for acquired radial nerve palsy. J Hand Surg Am 2007;32:526–530. 10.1016/j.jhsa.2007.01.012 17398364

[17] Bertelli JA. Nerve versus tendon transfer for radial nerve paralysis reconstruction. J Hand Surg Am 2020;45:418–426. 10.1016/j.jhsa.2019.12.009 32093993

